# Knowledge of and attitudes towards erosive tooth wear among students of two Chinese universities

**DOI:** 10.1186/s12903-020-01105-7

**Published:** 2020-04-15

**Authors:** Deng-wei Hong, Xiu-jiao Lin, Annette Wiegand, Hao Yu

**Affiliations:** 1 Fujian Key Laboratory of Oral Diseases & Fujian Provincial Engineering Research Center of Oral Biomaterial & Stomatological Key Laboratory of Fujian College and University, School and Hospital of Stomatology, Fujian Medical University, Fuzhou, China; 2 Department of Prosthodontics & Research Center of Dental Esthetics and Biomechanics, Fujian Medical University, Fuzhou, China; 3 Department of Preventive Dentistry, Periodontology and Cariology, University Medical Center Göttingen, Göttingen, Germany; 4 Department of Applied Prosthodontics, Graduate School of Biomedical Sciences, Nagasaki University, Nagasaki, Japan

**Keywords:** Erosive tooth wear, Knowledge, Attitude

## Abstract

**Background:**

To assess the knowledge of and attitudes towards erosive tooth wear among dental, medical, and non-medical university students of two Chinese universities.

**Methods:**

A questionnaire containing 15 questions on knowledge of erosive tooth wear and 10 questions on attitudes towards erosive tooth wear was designed, and its psychometric properties (reliability and validity) were analysed in a pilot study (*n* = 120 students). The following 3 groups of university students (*n* = 635) were recruited based on a convenience sampling technique and were distributed the questionnaire via an online survey system: dental students (DSs), medical students (MSs), and non-medical students (NSs). Differences in the scores between groups and genders were analysed by one-way analysis of variance (ANOVA). The Pearson correlation coefficient was used to determine the association between the knowledge and attitude scores (*P* < 0.05).

**Results:**

The questionnaire was found to be reliable, valid and reproducible. A total of 435 students participated in this study (response rate: 69.6%). The knowledge score of the DSs (11.5 ± 3.4) was significantly higher than those of the NSs (5.5 ± 4.0) and MSs (6.1 ± 4.0) (*P* < 0.001). The attitude score of the DSs (45.2 ± 6.5) was significantly higher than those of the NSs (41.1 ± 6.9) and MSs (41.8 ± 6.4) (*P* < 0.001). The majority of DSs expressed attitudes that were more accurate and positive than those expressed by the other 2 groups. The attitude score was positively correlated with the knowledge score (r = 0.237, *P* < 0.001).

**Conclusions:**

Dental students had more accurate knowledge of and more positive attitudes towards erosive tooth wear than medical and non-medical students. In this population, a positive correlation was established between knowledge of and attitudes towards erosive tooth wear.

## Background

Erosive tooth wear, defined as an irreversible loss of hard tissues due to a chemical process without the involvement of microorganisms, has become a topic of concern for both clinicians and researchers [1, 2]. Erosive tooth wear is a multifactorial disease that can be caused by intrinsic (e.g., gastric reflux and excessive vomiting) and/or extrinsic (e.g., acidic foods and drinks and acid fumes at work) factors. Early erosion causes no clinical discoloration or patient symptoms. However, in advanced stages, erosion often leads to the loss of dental hard tissues and the widespread exposure of dentin, resulting in tooth hypersensitivity, loss of occlusal vertical height and the destruction of pulp [1]. The prevalence of erosive tooth wear is increasing steadily, especially in young people [3, 4]. The worldwide prevalence of erosive tooth wear is estimated to be 30.4% for children and adolescents aged 8 to 19 years [5]. As the largest developing country, China has begun to pay more attention to erosive tooth wear, but prevalence data remain scarce. A survey in Guangzhou reported that 27.3% of 12- to 13-year-old children had at least one tooth with signs of erosive tooth wear [6]. Nearly half of all university students [7] and three-fourths of 12-year-olds [8] in Hong Kong were reported to have signs of erosive tooth wear.

The Chinese government recently released a series of health policies, including the Healthy China 2030 blueprint, the 13th Five-year Plan (2016–2020), and the Chronic Diseases Program in 2017, which all include the promotion of oral health [9]. In fact, the high prevalence of erosive tooth wear in China is thought to be related to the neglect of oral diseases and limited resources, poor knowledge and negative attitudes related to oral health [9]. However, information on public knowledge of and attitudes towards erosive tooth wear is lacking in China, while a limited number of studies have been performed worldwide. In a previous cross-sectional study, gaps were found in young adults’ knowledge of erosive tooth wear in the Netherlands. Knowledge of erosive tooth wear depended on individuals’ educational levels and dental information received in the past [10]. Chu et al. [11] reported a low level of awareness and knowledge of erosive tooth wear among adults aged 25 to 45 years in Hong Kong. A knowledge gradient regarding erosive tooth wear has been identified, with dental professionals having the most knowledge, followed by healthcare professionals and then laypersons [12]. However, the knowledge level of dental professionals is not as high as expected. Based on reports from Brazil, the United Kingdom, and Yemen, dental professionals exhibited insufficient knowledge of erosive tooth wear [12–14], which highlights the urgent need to improve education on erosive tooth wear worldwide. In addition, relatively little is known about the public attitudes towards erosive tooth wear. Skudutyte-Rysstad et al. [15] reported that adults with erosive tooth wear were more likely to have a low level of positive attitudes towards acidic drink consumption and were more reluctant to change than adults without erosion.

Therefore, the purpose of this study was to design a valid and reliable questionnaire to evaluate the knowledge levels and attitude of Chinese university students. The following null hypotheses were tested: 1) no differences would be found in the knowledge and attitude scores among university students with different majors; 2) no differences would be found in the knowledge and attitude scores between genders; and 3) no correlation would be found between Chinese university students’ knowledge of and attitudes towards erosive tooth wear.

## Methods

### Questionnaire development

A review of the medical and dental literature was undertaken to develop an appropriate questionnaire to evaluate knowledge of and attitudes towards erosive tooth wear. An item (question) pool including 63 questions was generated based on the literature [10, 13–17] (Supplementary Table 1). After a group discussion with 1 statistician, 2 dental specialists and 10 laypersons, 25 items were included in the questionnaire. The questionnaire was developed in English, translated into Chinese by a native English-speaking bilingual translator and then revised by a native Chinese-speaking bilingual translator. The questionnaire was then back-translated and verified with the original English questionnaire by another native Chinese-speaking bilingual person. The face validity of the questionnaire was confirmed by 2 senior experts in the field of preventive dentistry. Essential revisions of the included items were made based on feedback from the consultation [18].

Ethical approval from the local university was granted before the data collection took place (Ethical approval no. 2016Y9021). A pilot study was then undertaken with 120 university students to evaluate the psychometric properties of the questionnaire, including its reliability and validity. Reliability refers to the stability and internal consistency of a questionnaire. Validity refers to whether a questionnaire measures what it purports to measure [19].

The questionnaire included demographic questions concerning gender, age, and professional discipline. The remainder of the questionnaire was then divided into 2 sections to assess knowledge and attitudes (Supplemental Table 2). The knowledge section of the questionnaire included 15 true/false/don’t know questions on knowledge of erosive tooth wear (items K1-K15). The participants were asked to respond to each question with “true”, “false”, or “don’t know”. Each correct response received a score of 1, while an incorrect or “don’t know” response received a score of 0. The knowledge scores, which ranged from 0 to 15, were calculated by summing the scores for the items in the knowledge section. The knowledge score described the respondent’s knowledge of erosive tooth wear; higher sum scores indicated more accurate knowledge.

The attitude section of the questionnaire collected information using 10 positively framed statements (items A1-A10). The attitude score was based on a 5-point Likert scale, which is an instrument widely used in research on opinions, beliefs, and attitudes [19]. For the five response options, items were assigned 1 point for “strongly disagree”, 2 points for “disagree”, 3 points for “neither agree nor disagree”, 4 points for “agree”, and 5 points for “strongly agree”. The attitude scores, which ranged from 10 to 50, were calculated by summing the scores for the items in the attitude section of the questionnaire. The attitude score described the respondent’s attitude towards erosive tooth wear; higher scores indicated a more positive attitude.

### University students’ knowledge of and attitudes towards erosive tooth wear

After the psychometric properties of the questionnaire had been assessed, the questionnaire was revised and used to evaluate knowledge of and attitudes towards erosive tooth wear among Chinese university students. This cross-sectional study was carried out in 2 major universities (Fuzhou University and Fujian Medical University) in Fujian Province, China. The questionnaire was distributed to university students in their 4th and 5th academic years with different professional disciplines (majors): dental students (DSs), medical students (MSs), and non-medical students (NSs). There were ~ 36,000 undergraduates at the 2 universities in 2019, of whom ~ 8000 were 4th- and 5th-year students. Convenience sampling was used in the present study; the sample size was calculated based on a formula by Cochran stating that for a population of 8000, the approximate sample size should be 367 with a margin of error = 0.05 and a critical value of 1.96 [20]. Based on the response rates for dental erosion surveys reported in previous studies (45–79%) [11, 13, 15], a sample of 600 university students was considered sufficient. As a result, 198 DSs, 221 MSs, and 206 NSs were recruited from the School of Stomatology, the Medical School, and the Schools of Economics and Engineering, respectively. The students were invited to participate in this study at the end of the 2018–2019 academic year. A cover letter explaining the study design, the consent form, and the questionnaire was sent to the students using an online survey system (http://www.wjx.cn). The participation of the students was entirely voluntary, and the data were anonymously collected and analyzed, as the participants were not requested to provide their names or any other information that could be used to personally identify them.

### Statistical analysis

Cronbach’s alpha and the corrected item-total correlation (CITC) were employed to examine the internal consistency of the questionnaire. An alpha of 0.65–0.70 is considered minimally acceptable for research, an alpha of 0.70–0.80 is considered respectable, and an alpha of 0.80–0.90 is considered good [21]. Items with a CITC value < 0.3 should be deleted from a questionnaire [22]. The test-retest reliability was used to assess the temporal stability of the questionnaire; a test-retest correlation of 0.7–0.8 is considered acceptable, and a correlation of 0.8–0.9 is considered good [22]. Finally, exploratory factor analysis with varimax rotation was used to investigate the dimensionality of the questionnaire [23]. Bartlett’s test of sphericity was used to assess the relationship among variables, and the Kaiser-Meyer-Olkin (KMO) measure of sampling accuracy was used to assess the factorability of the correlation matrix. Scree plots were used to corroborate decisions regarding factor extraction [21].

The levels of knowledge and attitudes were defined based on Bloom’s original cut-off points [24]: scores that were over 80% of the total score indicated a high level of knowledge or a positive attitude; scores that were 60–80% of the total score indicated a moderate level of knowledge or a neutral attitude; and scores that were below 60% of the total score indicated a low level of knowledge or a negative attitude. Therefore, knowledge scores < 9, between 9 and 12, and > 12 indicated weak, moderate, and high levels of knowledge of erosive tooth wear, respectively. Attitude scores < 34, between 34 and 42, and > 42 indicated negative, neutral, and positive attitudes towards erosive tooth wear, respectively.

The demographic data were subjected to a descriptive statistical analysis. The knowledge and attitude scores were compared using one-way analysis of variance (ANOVA) and the least significant difference (LSD) test. An independent t-test was conducted to compare the knowledge and attitude scores between genders. The correlation between the knowledge and attitude scores was evaluated using Pearson’s correlation coefficient. All data were computerized and analyzed using the SPSS statistical software package for Windows 20.0 (IBM SPSS 20.0; SPSS, Chicago, IL, USA). Differences were considered statistically significant when the *P*-values were < 0.05.

## Results

### Psychometric properties of the questionnaire

The questionnaire was considered to be easily understandable, and all items were considered to be essential. The Cronbach’s alpha for the knowledge and attitude questionnaire sections were 0.83 and 0.88, respectively, indicating good internal consistency of the questionnaire. The CITC values were higher than 0.3 for all questions; thus, no item was deleted from the questionnaire. The test-retest correlation was considered good (r = 0.85 for the knowledge section and r = 0.91 for the attitude section, P all < 0.001) (Supplementary analysis 1 and 2).

For the attitude section of the questionnaire, the KMO value was 0.708, and Bartlett’s test of sphericity chi-square was 474.973 (*P* < 0.001). The exploratory factor analysis resulted in the emergence of 3 factors, namely, “etiology/causes”, “risk factors”, and “prevention and treatment”. For the attitude section of the questionnaire, the KMO value was 0.844, and Bartlett’s test of sphericity chi-square was 628.935 (*P* < 0.001). The exploratory factor analysis resulted in the emergence of 3 factors, namely, “attention degree”, “willingness to know”, and “willingness to change”. All 25 items in the questionnaire had satisfactory loadings (Supplementary analysis 1 and 2) [23]. Based on the results, the developed questionnaire was valid, reliable, and reproducible, and it was identified as a satisfactory tool for gathering the relevant data.

### University students’ knowledge and attitudes towards erosive tooth wear

Of the 625 questionnaires distributed to the students, 435 were returned, yielding a response rate of 69.6%. Among the 435 students, there were 141 NSs (32.2%), 140 DSs (32.2%), and 154 MSs (35.4%). Table 1 shows the age and gender distribution of the participants.

**Table 1:** Respondents’ demographic characteristics

Characteristics		Age (Mean ± SD)	Knowledge score (Mean ± SD)	*P*-value	Attitude score (Mean ± SD)	*P*-value
Gender	Male (*n* = 192)	22.5 ± 1.6	7.6 ± 4.7	0.825	41.7 ± 7.6	< 0.001 (Female>Male^a^)
	Female (*n* = 256)	22.2 ± 1.7	7.6 ± 4.7		43.5 ± 6.0	
Group	NSs (*n* = 141)	22.7 ± 1.5	5.5 ± 4.0	< 0.001 (DSs > NSs, MSs^a^)	41.1 ± 6.9	< 0.001 (DSs > NSs, MSs^a^)
	DSs (*n* = 154)	22.5 ± 1.2	11.5 ± 3.4		45.2 ± 6.5	
	MSs (*n* = 140)	223 ± 1.6	6.1 ± 4.0		41.8 ± 6.4	

*NSs* non-medical students, *DSs* dental students, *MSs* medical students

^a^>indicates statistical significance

**Fig. 1:**
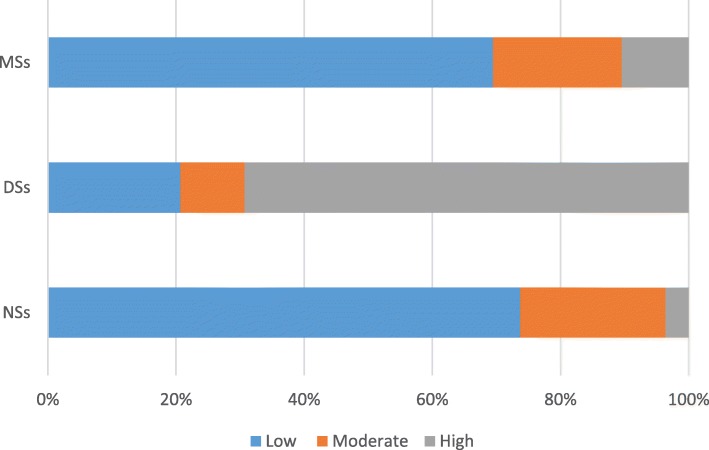
Distribution of the knowledge levels of the different groups. Knowledge scores < 9, between 9 and 12, and > 12 indicated weak, moderate, and high levels of knowledge of erosive tooth wear, respectively. *NSs = non-medical students, DSs = dental students, MSs = medical students

The knowledge score of the DSs was significantly higher than those of the NSs and MSs (*P* < 0.001). DSs had the highest scores for all items in the knowledge section of the questionnaire, while the other 2 groups showed similar scores (Table 2). Additionally, 69.3% of participants in the DS group, 3.5% in the NS group and 10.4% in the MS group exhibited high levels of knowledge (Fig. 1). Furthermore, no significant difference in the knowledge score between male and female students was found (*P* = 0.825).

**Table 2:** Comparison of the knowledge scores of non-medical, dental, and medical students

Item no.	Item content	NSs	DSs	MSs	*P*-value
K1	Erosive tooth wear is a form of cavities and tooth decay	0.15 ± 0.36	0.99 ± 0.08	0.12 ± 0.32	< 0.001 (DSs > NSs, MSs^a^)
K 2	Erosive tooth wear is caused by bacteria	0.16 ± 0.36	0.77 ± 0.42	0.12 ± 0.32	< 0.001 (DSs > NSs, MSs^a^)
K 3	Erosive tooth wear is an irreversible disease	0.28 ± 0.45	0.69 ± 0.47	0.29 ± 0.46	< 0.001 (DSs > NSs, MSs^a^)
K 4	One leading cause of tooth wear is acid in our food and drinks	0.47 ± 0.50	0.81 ± 0.39	0.47 ± 0.50	< 0.001 (DSs > NSs, MSs^a^)
K 5	Saliva is one of the most important defence mechanisms against erosion	0.30 ± 0.46	0.69 ± 0.47	0.36 ± 0.48	< 0.001 (DSs > NSs, MSs^a^)
K 6	Erosive tooth wear can occur if you often work in acidic environments	0.39 ± 0.49	0.81 ± 0.40	0.44 ± 0.50	< 0.001 (DSs > NSs, MSs^a^)
K 7	Erosive tooth wear can occur if you often have to vomit	0.43 ± 0.50	0.87 ± 0.34	0.50 ± 0.50	< 0.001 (DSs > NSs, MSs^a^)
K 8	Brushing your teeth immediately after consuming acidic food or drinks may make erosive tooth wear worse	0.21 ± 0.41	0.59 ± 0.49	0.22 ± 0.42	< 0.001 (DSs > NSs, MSs^a^)
K 9	Drinking before going to bed is a risk factor for developing erosive tooth wear	0.49 ± 0.50	0.76 ± 0.43	0.57 ± 0.50	< 0.001 (DSs > NSs, MSs^a^)
K 10	Drinking immediately after strenuous exercise increases a person’s risk for erosive tooth wear	0.47 ± 0.50	0.66 ± 0.48	0.48 ± 0.50	< 0.001 (DSs > NSs, MSs^a^)
K 11	Erosive tooth wear may lead to pain and sensitivity	0.62 ± 0.49	0.88 ± 0.33	0.71 ± 0.46	< 0.001 (DSs > NSs, MSs^a^)
K 12	Erosive tooth wear can lead to the progressive loss of the surface of the tooth	0.46 ± 0.50	0.84 ± 0.37	0.61 ± 0.49	< 0.001 (DSs > MSs > NSs^a^)
K 13	Drinking a whole bottle of soda in several sittings rather than in just one sitting decreases a person’s risk for erosive tooth wear	0.28 ± 0.45	0.66 ± 0.48	0.30 ± 0.46	< 0.001 (DSs > NSs, MSs^a^)
K 14	Using a fluoride toothpaste will prevent erosive tooth wear	0.40 ± 0.49	0.71 ± 0.46	0.51 ± 0.50	< 0.001 (DSs^a^NSs, MSs^a^)
K 15	Using a straw when you drink soda may help avoid erosive tooth wear	0.42 ± 0.50	0.72 ± 0.45	0.40 ± 0.49	< 0.001 (DSs > NSs, MSs^a^)

*NSs* non-medical students, *DSs* dental students, *MSs* medical students

^a^>indicates statistical significance

**Fig. 2:**
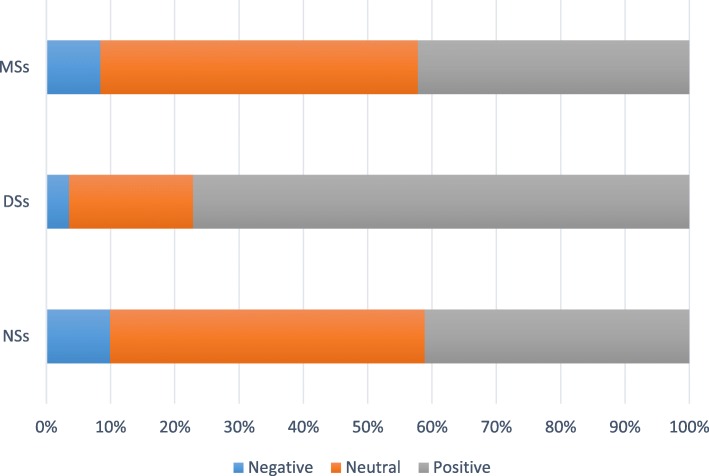
Distribution of the attitude levels of the different groups. Attitude scores < 34, between 34 and 42, and > 42 indicated negative, neutral, and positive attitudes towards erosive tooth wear, respectively. *NSs = non-medical students, DSs = dental students, MSs = medical students

The attitude score of the DSs was significantly higher than those of the NSs and MSs (*P* < 0.001). DSs had the highest scores for all items in the attitude section of the questionnaire, while the other 2 groups had similar scores for the majority of questions (Table 3). Additionally, the majority of participants in the DS group (77.1%) expressed positive attitudes, while only 41.4% in the NS group and 42.2% in the MS group exhibited positive attitudes (Fig. 2). Furthermore, there was a significant difference in the attitude score between male and female students (*P* < 0.001). Finally, the attitude score was positively correlated with the knowledge score (r = 0.237, P < 0.001).

**Table 3:** Comparison of the attitude scores of non-medical, dental and medical students

Item No.	Contents	NSs	DSs	MSs	P-value
A1	I think oral health is just as important as general health	4.38 ± 0.90	4.64 ± 0.92	4.47 ± 0.69	0.032 (DSs > NSs; MSs > NSs^a^)
A2	I think prevention is better than a cure	4.39 ± 0.81	4.66 ± 0.90	4.51 ± 0.73	0.023 (DSs, MSs > NSs^a^)
A3	It is essential to visit a dentist at least every half year for a regular dental check-up	4.09 ± 0.96	4.66 ± 0.89	4.32 ± 0.77	< 0.001 (DSs > NSs, MSs^a^)
A4	I would think that it is bad if I learned that my teeth had been damaged by acid	4.43 ± 0.80	4.61 ± 0.80	4.51 ± 0.68	0.133
A5	It is worth spending more time and energy on studying knowledge about erosive tooth wear	3.97 ± 0.92	4.57 ± 0.80	4.09 ± 0.89	< 0.001 (DSs > NSs, MSs^a^)
A6	I am concerned with whether or not drinks I consume are acidic	3.82 ± 1.01	4.27 ± 0.97	3.80 ± 0.97	< 0.001 (DSs > NSs, MSs^a^)
A7	I am concerned with whether or not a toothpaste contains fluoride	3.74 ± 1.07	4.23 ± 1.03	3.73 ± 1.00	< 0.001 (DSs > NSs, MSs^a^)
A8	To prevent erosive tooth wear, I would change my dietary habits (such as controlling my consumption of soft drinks)	4.09 ± 0.85	4.49 ± 0.88	4.14 ± 0.82	< 0.001 (DSs > NSs, MSs^a^)
A9	To prevent erosive tooth wear, I would change my behavior habits (such as drinking from a straw)	4.09 ± 0.81	4.53 ± 0.74	4.18 ± 0.79	< 0.001 (DSs > NSs, MSs^a^)
A10	I would see a doctor immediately if I learned that my teeth had been damaged by acid	4.07 ± 0.85	4.54 ± 0.77	4.08 ± 0.91	< 0.001 (DSs > NSs, MSs^a^)

*NSs* non-medical students, *DSs* dental students, *MSs* medical students

^a^>indicates statistical significance

## Discussion

Based on the present findings, the null hypotheses that no differences would be found in the knowledge and attitude scores among students with different majors, that no difference would be found in the attitude score between genders and that no correlation would be found between the knowledge and attitude scores were rejected. The null hypothesis that no difference would be found in the knowledge score between genders was accepted.

Erosive tooth wear may contribute to pain and sensitivity, and it may also impose functional and aesthetic limitations [1]. However, access to the optimal management of erosive tooth wear is difficult due to escalating costs and limited resources, especially in developing countries [25]. In most cases, erosive tooth wear is detected at late stages, and the lack of public knowledge of erosive tooth wear has been at least partly attributed to delays in diagnosis and treatment [7, 8]. A lack of knowledge and negative attitudes might constitute potential barriers to effectively controlling and preventing erosive tooth wear [26, 27]. However, limited surveys have been performed to investigate knowledge of and/or attitudes towards erosive tooth wear. More importantly, although psychometric evaluations, including item analysis, principal component analysis, reliability, and validity are considered the essential elements in designing and developing a questionnaire [4], the psychometric qualities of the questionnaires used in previous studies have not been tested/reported [10, 13, 14]. Failure to sufficiently develop a questionnaire may lead to the misinterpretation of the results [22]. Therefore, the development process of this questionnaire was presented in sufficient detail to enable practitioners, educators, and policy makers to make evidence-based decisions about whether to apply the findings. In this study, the psychometric properties of the questionnaire were assessed using a systematic approach and the result was satisfactory. This instrument is considered valuable for comparing knowledge levels and attitudes with regard to erosive tooth wear across countries or studies.

Although the majority of the participants agreed or strongly agreed that prevention is better than a cure, there was wide variation in knowledge and attitudes among university students with different majors. DSs exhibited significantly greater knowledge of and more positive attitudes towards erosive tooth wear than the other 2 types of students. As the future oral health authorities of society, DSs are expected to possess accurate knowledge of and express positive attitudes towards erosive tooth wear, as DSs with such knowledge and attitudes will be more likely to improve the oral health status of their patients. However, 30.7% of DSs exhibited moderate or low knowledge levels, and 22.9% of the DSs expressed neutral or negative attitudes. In the DS group, the 2 items in the knowledge section of the questionnaire with the lowest scores were K10 (“brushing your teeth immediately after consuming acidic food or drinks may make erosive tooth wear worse”), which was incorrectly answered by 57 individuals (40.7%), and K12 (“drinking immediately after strenuous exercise increases a person’s risk for erosive tooth wear”), which was incorrectly answered by 48 individuals (34.3%). These findings indicated that Chinese DSs lacked an understanding of the risk factors for erosive tooth wear. Dental subjects are covered predominantly in the 3rd and 4th years of undergraduate education in China, resulting in excessive levels of academic stress and depression [28]. Moreover, although the subject of erosive tooth wear is included in the curriculum of most Chinese dental schools, it is not considered a core topic, which is similar to the U.S. and Canada [29]. The abovementioned facts may contribute to a lack of understanding of the risk factors of erosive tooth wear among Chinese DSs. The present study highlighted the urgent need for Chinese DSs to receive systematic education on erosive tooth wear.

Oral health is an indispensable component of systemic health [30, 31]. Erosive tooth wear has been reported to be closely related to some systemic diseases, such as gastroesophageal reflux disease, high blood pressure, salivary gland agenesis, and Sjögren’s syndrome [1, 16, 32, 33]. This comorbidity often requires joint efforts by both dentists and physicians to identify the best treatment for each patient. In addition, as the major providers of health services, physicians are expected to play an important role in public oral health promotion. In the present study, significant differences in the scores for K3 (“erosive tooth wear can lead to the progressive loss of the surface of the tooth”) and A10 (“it is essential to visit a dentist at least every half a year for a regular dental check-up”) between the MSs and NSs were found. These results suggested that the MSs had a better understanding of the consequences of erosive tooth wear and were more willing to have a regular check-up to prevent erosive tooth wear. However, due to inadequate knowledge, MSs may lack the skills needed to play an active role in preventing erosive tooth wear [34]. No significant differences in the knowledge and attitude scores between the MSs and NSs were found. The majority of NSs and MSs exhibited low levels of knowledge of and moderate attitudes towards erosive tooth wear. The MSs and NSs even considered erosive tooth wear to be a form of cavities and tooth decay (86.8%) and believed that it is caused by bacteria (80.4%), which was consistent with a previous report in Hong Kong [11]. These findings indicated that the knowledge and attitudes of Chinese MSs were inadequate. In China, dentistry has been regarded as a major that is independent of general medicine, resulting in the neglect of oral health in general medical education [28]. Similar to MSs in other countries, Chinese MSs receive very limited training in the treatment of oral health problems and the maintenance of good oral health [35, 36]. Oral health education is an effective means to promote oral health by providing information to improve oral health knowledge and attitudes [37]. In fact, physicians and medical providers are willing to acquire oral health knowledge and to practice the necessary skills. However, they receive too little oral health education and training [38, 39]. Given that teaching in schools can effectively improve oral health knowledge [40, 41], the need to integrate erosive tooth wear in university curricula is evident.

Almost all participants (92.9%) were concerned with damage to their teeth from acids, indicating a more positive attitude than that found in a previous study carried out in Norway [15]. For all 3 investigated groups, the 2 items in the attitude section of the questionnaire with the lowest scores were A6 (“I am concerned with whether or not the drinks I consume are acidic”) and A7 (“I am concerned with whether or not a toothpaste contains fluoride”). Reducing dietary acid intake can be key to delaying the progression of erosive tooth wear [15]. Using fluoride toothpaste is the most economical and simplest way to prevent erosive tooth wear [17]. However, it seemed that Chinese university students paid little attention to whether their diets contained acid or whether the toothpaste they used contained fluoride, which is also consistent with previous studies [10, 15].

The female students showed more positive attitudes towards erosive tooth wear than the male students. This finding was consistent with previous studies that found that females had better health attitudes than their male colleagues [27, 42]. The difference between genders may be explained by males’ habitual personal neglect and females’ stronger concerns with their body and appearance [43]. Moreover, females tend to have more interest in health than males [44]. Finally, in this population, attitudes were positively correlated with knowledge of erosive tooth wear. Knowledge is thought to be one of the most important factors involved in forming and changing attitudes [7, 15, 45]. Therefore, improving the knowledge of university students may contribute to changes in their attitudes towards erosive tooth wear. However, further studies are needed to confirm this hypothesis.

This cross-sectional study was carried out at the two major universities in Fujian Province, China. Although the included students came from different parts of China, the results of this survey may not be generalizable to the whole country. Moreover, because all participants were university students of similar ages, the effects of age on knowledge of and attitude towards erosive tooth wear were not investigated in the present study. Although the response rate of this survey was relatively low, it was within the range reported in previous studies [11, 13, 15]. The low response rate worldwide may be attributed to public ignorance of erosive tooth wear. Moreover, although no consensus has been reached on the treatment of Likert scale score data, the Likert scale scores were treated as interval data as described in previous studies [46, 47]. However, importantly, the treatment of single Likert scale items as numerical data is debatable. The data may be skewed and the items may not capture the true limits of the respondents’ attitude, which is considered a limitation of the present study. Despite these limitations, the results provide important information that could serve as a basis for future planning to enhance Chinese university students’ knowledge of and attitudes towards erosive tooth wear.

## Conclusions

Within the limitations of the present study, the following conclusions were drawn:

1. Dental students had more accurate knowledge of and more positive attitudes towards erosive tooth wear than medical and non-medical students.
2. No significant difference in the level of knowledge of erosive tooth wear between genders was found. However, female students showed more positive attitudes towards erosive tooth wear.
3. Attitudes were significantly and positively correlated with knowledge of erosive tooth wear.

## Supplementary information

**Additional file 1 : Supplementary Table 1**: Questionnaire used in this study.

**Additional file 2 : Supplementary Table 2**: Initial items collected for the development of questionnaire.

**Additional file 3 : Supplementary Analysis 1**: Psychometric properties of the knowledge questionnaire.

**Additional file 4 : Supplementary Analysis 2**: Psychometric properties of the attitude questionnaire.

## Data Availability

Further data may be requested by contacting the corresponding author. Any data regarding the study will be willingly provided.

## Abbreviations

DSs: Dental students
MSs: Medical students
NSs: Non-medical students
CITC: Corrected item-total correlation
LSD: Least significant difference
ANOVA: Analysis of variance
