# Supplementary material for: Knowledge of and attitudes towards erosive tooth wear among students of two Chinese universities
Source: BMC Oral Health. 2020 Apr 15;20:110. doi: 10.1186/s12903-020-01105-7 (PMC7160986; doi:10.1186/s12903-020-01105-7)
Supplement: Supplementary file 1 — Additional file 1 : Supplementary Table 1: Questionnaire used in this study. [file 12903_2020_1105_MOESM1_ESM.docx]

**Supplementary Table 1.** Initial items collected for the development of questionnaire.

|  | Items | Source |
| --- | --- | --- |
| Knowledge section of the questionnaire | Additional preventive methods against dental erosion include the use of fluoridated tooth paste  Additional preventive measures against dental erosion may include wearing a night guard  Additional preventive measures against dental erosion may include stopping biting on hard objects  I think dental erosion can be caused by bruxism  I think dental erosion can be caused by acidic drinks  I think dental erosion can be caused by excessive teeth brushing with a hard tooth brush  Other causes of dental erosion include biting on hard objects such as nails, pens and nuts  Other causes of dental erosion include high sugar consumption  Other causes of dental erosion include systemic diseases (e.g., eating disorders, stomach reflux)  The main clinical sign of the early stage of dental erosion is shortening of teeth  The main clinical sign of the early stage of dental erosion is facial muscle tension  The main clinical sign of the early stage of dental erosion is the smoothening of enamel surfaces  To prevent dental erosion, you should reduce acidic drinks consumption | Contents adapted from Al-Ashtal [Al-Ashtal et al., 2015] |
|  | 14. Dental erosion is another name for tooth wear  15. Acids in your food and drinks can causes dental erosion  16. Teeth become shorter are the consequences of dental erosion  17. Fluoride in toothpaste makes teeth stronger  18. Brushing your teeth immediately after drinking orange juice causes more dental erosion  19. Keeping the fizzy drink in your mouth for a long time before you swallow is most likely to cause dental erosion  20. Drinking sports drinks during exercise poses the greatest risk of dental erosion  21. With a dry mouth, you get tooth wear faster  22. You have less chance of tooth wear when you drink a glass of water immediately after drinking cola  23. You have less chance of tooth wear if you drink milk instead of cola  24. Dental erosion can occur if you often have to vomit | Contents adapted from Verploegen [Verploegen, and Schuller, 2019] |
|  | 25. Sugar can contribute to tooth erosion  26. Reducing your intake of acidic drinks is recommended to prevent dental erosion  27. Reducing your intake of sugar is recommended to prevent dental erosion  28. The use of fluoride is recommended to prevent dental erosion  29. Reducing your intake of fruits is recommended to prevent dental erosion | Contents adapted from Hermont [Hermont et al., 2011] |
|  | 30. Dental erosion can lead to the progressive loss of the surface of the tooth  31. Dental erosion may lead to pain and sensitivity  32. Using a straw when you drink soda may help avoid dental erosion  33. Acidic medicines, such as chewable acetylsalicylic acid, effervescent vitamin C tablets and acidic saliva substitutes could have erosive potential  34. Alcoholics may be at particular risk of dental erosion and tooth wear  35. Chewing gum after a meal helps to reduce postprandial esophageal acid exposure | Contents based on Lussi A et al. [Lussi A et al., 2011] |
|  | 36. Saliva is one of the most important defence mechanisms for dental erosion  37. Erosion may be associated with a low salivary flow and a low buffering capacity | Contents based on Hara et al. [Hara et al., 2006] |
|  | 38. Drinking before going to bed is a risk factor for developing dental erosion  39. Dental erosion can occur if you often working in acidic environments  40. Reducing the frequency with which you consume acidic food and drinks may help avoid dental erosion  41. Using a straw when you drink soda may help avoid dental erosion  42. Use a soft toothbrush may help avoid dental erosion | Contents based on Amaechi et al. [Amaechi, and Higham, 2005] |
|  | 43. Dental erosion is a form of cavities and tooth decay  44. Dental erosion is caused by bacteria  45. Dental erosion is an irreversible disease | Contents based on experts in preventive dentistry |
| Attitude questionnaire | 46. I think I am aware of my teeth and oral health  47. It is not worth the trouble to change one′s habits  48. I am concerned about diet and health  49. I am not concerned with whether or not the drinks I consume are acidic  50. I try to limit how often I drink soft drinks, juice, etc.  51. I find it difficult to avoid drinking soft drinks, juice, etc.  52. I feel I am able to control my consumption of soft drinks, juice, etc.  53. It is not important to me whether or not my teeth have been damaged by acid  54. I would think that it is bad if I learned that my teeth had been damaged by acid  55. I would change my habits if I learned that my teeth have been damaged by acids | Contents adapted from Skudutyte-Rysstad [Skudutyte-Rysstad et al., 2013] |
|  | 56. I think oral health is just as important as general health  57. I think prevention is better than cure  58. It is essential to visit a dentist at least every half year for a regular checkup  59. It is worth spending more time and energy on studying knowledge about dental erosion  60. I am concerned with whether or not a toothpaste contains fluoride  61. To prevent dental erosion, I would change my dietary habits (such as control my consumption of soft drinks)  62. To prevent dental erosion, I would change my behavior habits (such as drinking from a straw)  63. I would see a doctor immediately if I learned that my teeth had been damaged by acid | Contents based on the experts in preventive dentistry |
