# Supplementary material for: Knowledge of and attitudes towards erosive tooth wear among students of two Chinese universities
Source: BMC Oral Health. 2020 Apr 15;20:110. doi: 10.1186/s12903-020-01105-7 (PMC7160986; doi:10.1186/s12903-020-01105-7)
Supplement: Supplementary file 2 — Additional file 2 : Supplementary Table 2: Initial items collected for the development of questionnaire. [file 12903_2020_1105_MOESM2_ESM.docx]

**Supplementary Table 2.** Questionnaire used in this study

| Section | Item no. | Item content |
| --- | --- | --- |
| knowledge of erosive tooth wear | K1 | Erosive tooth wear is a form of cavities and tooth decay |
|  | K 2 | Erosive tooth wear is caused by bacteria |
|  | K 3 | Erosive tooth wear is an irreversible disease |
|  | K 4 | One leading cause of tooth wear is acid in our food and drinks |
|  | K 5 | Saliva is one of the most important defence mechanisms against erosion |
|  | K 6 | Erosive tooth wear can occur if you often work in acidic environments |
|  | K 7 | Erosive tooth wear can occur if you often have to vomit |
|  | K 8 | Brushing your teeth immediately after consuming acidic food or drinks may make erosive tooth wear worse |
|  | K 9 | Drinking before going to bed is a risk factor for developing erosive tooth wear |
|  | K 10 | Drinking immediately after strenuous exercise increases a person’s risk for erosive tooth wear |
|  | K 11 | Erosive tooth wear may lead to pain and sensitivity |
|  | K 12 | Erosive tooth wear can lead to the progressive loss of the surface of the tooth |
|  | K 13 | Drinking a whole bottle of soda in several sittings rather than in just one sitting decreases a person’s risk for erosive tooth wear |
|  | K 14 | Using a fluoride toothpaste will prevent erosive tooth wear |
|  | K 15 | Using a straw when you drink soda may help avoid erosive tooth wear |
| Attitude towards erosive tooth wear | A1 | I think oral health is just as important as general health |
|  | A2 | I think prevention is better than a cure |
|  | A3 | It is essential to visit a dentist at least every half year for a regular dental check-up |
|  | A4 | I would think that it is bad if I learned that my teeth had been damaged by acid |
|  | A5 | It is worth spending more time and energy on studying knowledge about erosive tooth wear |
|  | A6 | I am concerned with whether or not drinks I consume are acidic |
|  | A7 | I am concerned with whether or not a toothpaste contains fluoride |
|  | A8 | To prevent erosive tooth wear, I would change my dietary habits (such as controlling my consumption of soft drinks) |
|  | A9 | To prevent erosive tooth wear, I would change my behavior habits (such as drinking from a straw) |
|  | A10 | I would see a doctor immediately if I learned that my teeth had been damaged by acid |
