# Supplementary material for: Knowledge of and attitudes towards erosive tooth wear among students of two Chinese universities
Source: BMC Oral Health. 2020 Apr 15;20:110. doi: 10.1186/s12903-020-01105-7 (PMC7160986; doi:10.1186/s12903-020-01105-7)
Supplement: Supplementary file 3 — Additional file 3 : Supplementary Analysis 1: Psychometric properties of the knowledge questionnaire. [file 12903_2020_1105_MOESM3_ESM.docx]

**Supplementary Analysis 1.** Psychometric properties of the knowledge questionnaire.

1. Reliability test

| Item no. | Corrected item-total correlation | Cronbach' s alpha | Test-retest reliability |
| --- | --- | --- | --- |
| K1 | 0.604 | 0.894 | 0.848 |
| K2 | 0.662 |  |  |
| K3 | 0.582 |  |  |
| K4 | 0.592 |  |  |
| K5 | 0.647 |  |  |
| K6 | 0.724 |  |  |
| K7 | 0.575 |  |  |
| K8 | 0.745 |  |  |
| K9 | 0.491 |  |  |
| K10 | 0.55 |  |  |
| K11 | 0.318 |  |  |
| K12 | 0.399 |  |  |
| K13 | 0.664 |  |  |
| K14 | 0.45 |  |  |
| K15 | 0.561 |  |  |

2. Scree plot


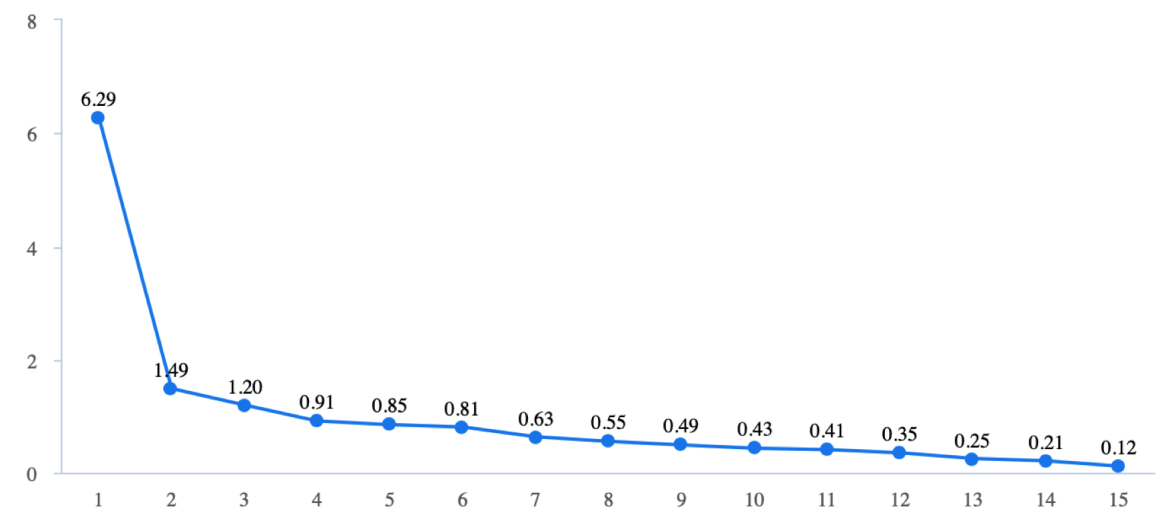


3. Rotated component matrix

| Item no. | Factor loading | |  | Communalities |
| --- | --- | --- | --- | --- |
|  | Factor 1 | Factor 2 | Factor 3 |  |
| K1 | 0.766 | -0.107 | -0.265 | 0.669 |
| K2 | 0.842 | 0.060 | -0.264 | 0.782 |
| K3 | 0.577 | 0.193 | 0.551 | 0.674 |
| K4 | 0.050 | 0.610 | 0.169 | 0.403 |
| K5 | 0.730 | 0.398 | 0.179 | 0.724 |
| K6 | 0.783 | 0.393 | 0.150 | 0.79 |
| K7 | -0.031 | 0.883 | 0.036 | 0.782 |
| K8 | 0.831 | 0.264 | -0.185 | 0.794 |
| K9 | 0.716 | 0.044 | 0.199 | 0.555 |
| K10 | 0.638 | 0.011 | 0.000 | 0.407 |
| K11 | 0.209 | 0.071 | -0.789 | 0.671 |
| K12 | 0.170 | 0.426 | 0.504 | 0.464 |
| K13 | 0.234 | 0.816 | 0.126 | 0.736 |
| K14 | 0.734 | 0.049 | 0.122 | 0.557 |
| K15 | 0.113 | 0.718 | -0.309 | 0.623 |
| Variance interpretation rate% | 33.792 | 19.719 | 10.695 | - |
| Cumulative variance interpretation rate% | 33.792 | 53.51 | 64.205 | - |
| Kaiser-Meyer-Olkin Measure of Sampling Adequacy | 0.708 | | | |
| Bartlett’s test chi-square value | 474.973 | | | |
| P-value | <0.001 | | | |
