# Supplementary material for: Knowledge of and attitudes towards erosive tooth wear among students of two Chinese universities
Source: BMC Oral Health. 2020 Apr 15;20:110. doi: 10.1186/s12903-020-01105-7 (PMC7160986; doi:10.1186/s12903-020-01105-7)
Supplement: Supplementary file 4 — Additional file 4 : Supplementary Analysis 2: Psychometric properties of the attitude questionnaire. [file 12903_2020_1105_MOESM4_ESM.docx]

**Supplementary Analysis 2.** Psychometric properties of the attitude questionnaire.

1. Reliability test

| Item no. | Corrected item-total correlation | Cronbach' s alpha | Test-retest reliability |
| --- | --- | --- | --- |
| A1 | 0.658 | 0.901 | 0.909 |
| A2 | 0.577 |  |  |
| A3 | 0.72 |  |  |
| A4 | 0.657 |  |  |
| A5 | 0.758 |  |  |
| A6 | 0.623 |  |  |
| A7 | 0.698 |  |  |
| A8 | 0.733 |  |  |
| A9 | 0.671 |  |  |
| A10 | 0.562 |  |  |

2. Scree plot


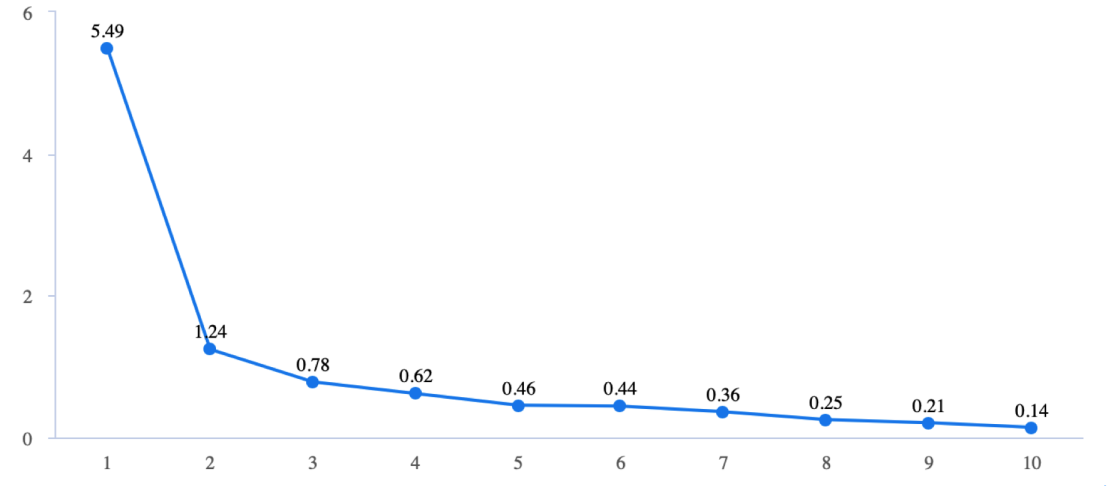


3. Rotated component matrix

| Item no. | Factor loading | |  | Communalities |
| --- | --- | --- | --- | --- |
|  | Factor 1 | Factor 2 | Factor 3 |  |
| A1 | 0.870 | 0.138 | 0.238 | 0.833 |
| A2 | 0.829 | 0.082 | 0.195 | 0.731 |
| A3 | 0.697 | 0.447 | 0.183 | 0.719 |
| A4 | 0.750 | 0.284 | 0.207 | 0.685 |
| A5 | 0.516 | 0.661 | 0.206 | 0.746 |
| A6 | 0.147 | 0.882 | 0.159 | 0.825 |
| A7 | 0.211 | 0.702 | 0.409 | 0.705 |
| A8 | 0.229 | 0.460 | 0.732 | 0.800 |
| A9 | 0.200 | 0.476 | 0.658 | 0.700 |
| A10 | 0.280 | 0.068 | 0.828 | 0.769 |
| Variance interpretation rate% | 29.96 | 24.569 | 20.613 | - |
| Cumulative variance interpretation rate% | 29.96 | 54.529 | 75.142 | - |
| Kaiser-Meyer-Olkin Measure of Sampling Adequacy | 0.844 | | | |
| Bartlett’s test chi-square value | 628.935 | | | |
| P-value | <0.001 | | | |
